# Surgical Site Infection and Antimicrobial Resistance After Mastectomy in Dogs and Cats: A 615‐Case Multicentre Cohort and Infection‐Control Audit

**DOI:** 10.1002/vms3.71322

**Published:** 2026-11-03

**Authors:** Mohammad Hedayatinia, Ferdos Gorjian, Mobina Pato, Mohammad Ali Maadi, Reza Ahmadi, Arman Abdous

**Affiliations:** ^1^ Department of Veterinary Medicine Shoushtar Branch Islamic Azad University Shoushtar Iran; ^2^ Department of Microbiology Faculty of Veterinary Medicine SR.C. Islamic Azad University Tehran Iran; ^3^ Department of Clinical Sciences School of Veterinary Medicine Shiraz University Shiraz Iran; ^4^ Department of Small Animal Internal Medicine Islamic Azad University, Garmsar Branch Semnan Iran; ^5^ Department of Epidemiology and Biostatistics School of Public Health Tehran University of Medical Sciences Tehran Iran

**Keywords:** cats, dogs, drug resistance, infection control, mastectomy, microbial, surgical wound infection

## Abstract

**Background:**

Surgical site infection (SSI) is an important complication after mastectomy and antimicrobial resistance may complicate treatment.

**Objectives:**

To estimate 30‐day SSI incidence after mastectomy in dogs (*Canis lupus familiaris* Linnaeus, 1758) and cats (*Felis catus* Linnaeus, 1758), evaluate patient‐ and procedure‐related factors, describe perioperative antimicrobial use and microbiology and assess clinic infection‐control practices.

**Methods:**

This multicentre retrospective cohort included 615 mastectomy procedures from 12 veterinary clinics in Tehran Province, Iran, between 2020 and 2025. SSI‐positive records were independently re‐reviewed by two investigators using contemporary veterinary SSI terminology. Clinic‐stratified conditional logistic regression evaluated seven clinically selected predictors.

**Results:**

SSI occurred after 54/615 procedures (8.8%; 95% CI, 6.8%–11.3%); 50 infections were superficial and four deep. Surgical duration per 30 min (aOR, 2.37; 95% CI, 1.61–3.49), intraoperative hypothermia (aOR, 3.66; 95% CI, 1.39–9.62), body condition score per point (aOR, 1.60; 95% CI, 1.05–2.42) and previous major surgery (aOR, 3.57; 95% CI, 1.49–8.55) were associated with SSI. An MDR isolate was identified in 16/33 cultured SSI cases and antimicrobial–isolate discordance was present in 15/30 assessable comparisons. Mean clinic infection‐control audit score was 4.6 ± 1.7/10.

**Conclusions:**

Longer surgery, intraoperative hypothermia, higher body condition score and previous major surgery were associated with SSI in this predominantly canine cohort of intact animals undergoing mastectomy without concurrent ovariohysterectomy. These associations were not independently demonstrated in cats. The findings support standardised surveillance, perioperative risk reduction, culture‐guided treatment and antimicrobial stewardship.

**Clinical trial registration:**

Not applicable. This was a retrospective observational study and clinic‐level audit and was not a clinical trial.

## Graphical abstract

In 615 mastectomy procedures in intact dogs and cats without concurrent ovariohysterectomy, 54 surgical site infections (8.8%) were identified during 30‐day surveillance. Longer surgery, intraoperative hypothermia and previous major surgery showed the most consistent adjusted associations, predominantly reflecting dogs; 16/33 cultured infections involved multidrug‐resistant organisms and 15/30 assessable antimicrobial–isolate comparisons were discordant. Associations were not independently demonstrated in cats; the findings support standardised surveillance, perioperative risk reduction, culture‐guided treatment and antimicrobial stewardship.

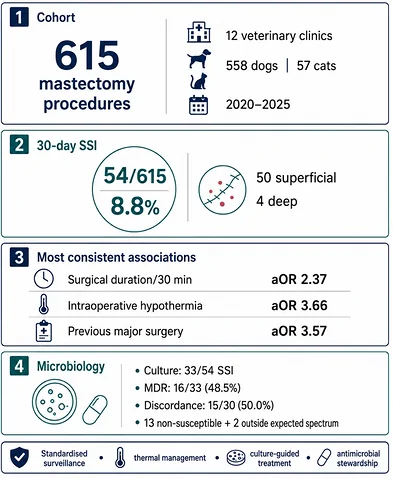

## 1 Introduction

Mammary tumours are common in female dogs (*Canis lupus familiaris* Linnaeus, 1758) and are also clinically important in cats (*Felis catus* Linnaeus, 1758), in which a high proportion of mammary tumours are malignant (Cassali et al. 2025; Rodrigues‐Jesus et al. 2025). Mastectomy may range from removal of a single gland to regional, radical, or bilateral procedures, with surgical extent influencing tissue dissection and postoperative morbidity (Spåre et al. 2021; Fuertes‐Recuero et al. 2025).

Surgical site infection (SSI) can delay healing and result in antimicrobial treatment, readmission, or reoperation. The recent veterinary consensus defines SSI according to its relationship to the surgical intervention and the anatomical tissues involved and distinguishes superficial incisional, deep incisional and organ/space SSI independently of the surveillance interval (Verwilghen et al. 2026). Recent veterinary studies and reviews have identified operative or anaesthetic duration, procedural extent, body weight, intraoperative hypothermia and American Society of Anesthesiologists (ASA) physical status as relevant candidate factors in selected surgical populations (Stetter et al. 2021; Fuertes‐Recuero et al. 2025; Cavalli et al. 2025, 2026).

Current antimicrobial‐stewardship guidance provides a framework for interpreting surgical antimicrobial prophylaxis (SAP). The European Network for Optimisation of Veterinary Antimicrobial Therapy (ENOVAT) recommends against routine perioperative and postoperative SAP for clean soft‐tissue procedures other than neutering, while its supporting systematic review found generally trivial or small estimated benefits across the surgical categories evaluated (Allerton et al. 2026; Sørensen et al. 2026). Recent clinical studies similarly question routine prolonged postoperative prophylaxis in many clean or elective procedures (Spåre et al. 2021; Paeckel et al. 2024; Di Filippo et al. 2025). The International Society for Companion Animal Infectious Diseases–World Small Animal Veterinary Association (ISCAID–WSAVA) targets recommend that, by 2030, at least 95% of clean procedures receive no prophylactic antimicrobials and fewer than 5% of surgical patients are discharged with antimicrobials (ISCAID and WSAVA 2025).

Antimicrobial resistance further supports culture‐guided treatment once SSI develops. Resistant gram‐positive and gram‐negative organisms occur in companion‐animal wounds (Danieli et al. 2026) and household sharing of resistant organisms, although generally uncommon, is more frequent when infection is present (Muturi et al. 2026). Molecular investigations have also documented sharing of methicillin‐resistant *Staphylococcus pseudintermedius* (MRSP) between an infected dog and human contacts (de Menezes et al. 2026).

The objectives of this study were to determine 30‐day SSI incidence and severity after mastectomy; evaluate patient‐, pathology‐ and procedure‐related associations; describe antimicrobial use, microbiology and resistance; and characterise clinic infection‐control practices.

## 2 Materials and Methods

### 2.1 Ethical Considerations

The study comprised a retrospective review of de‐identified clinical records and a clinic‐level infection‐control audit; no experimental treatment or prospective intervention was performed. Owner‐identifying information was not transferred to the analytical dataset, telephone follow‐up was conducted by clinic personnel using existing records and clinic managers permitted the audit.

### 2.2 Study Design and Reporting

The patient‐level component was a multicentre retrospective cohort and the facility‐level component was a cross‐sectional descriptive audit. The analytical unit was the mastectomy procedure. Each included animal contributed one eligible mastectomy procedure; therefore, the 615 procedures represented 615 unique animals. Reporting followed the strengthening the reporting of observational studies in epidemiology (STROBE) statement and its veterinary extension, STROBE‐Vet (von Elm et al. 2007; Sargeant et al. 2016); the completed checklist is provided in Supplementary Table S1.

### 2.3 Setting

Records were obtained from 12 veterinary hospitals and clinics in Tehran Province, Iran, for procedures performed from 1 January 2020 through 31 December 2025. The infection‐control audit was a one‐time facility assessment and was treated as contextual descriptive information rather than as a contemporaneous causal exposure.

### 2.4 Eligibility Criteria

Records were eligible when a dog or cat underwent simple, regional, radical, or bilateral mastectomy and sufficient information was available to determine the procedure and 30‐day wound outcome. Records were excluded when more than 20% of key demographic, surgical and outcome variables were missing; when the 30‐day outcome could not be established; when a concurrent major procedure such as ovariohysterectomy or splenectomy was performed; when infection involved the surgical field at the time of mastectomy; when preoperative chemotherapy or radiotherapy had been administered; or when euthanasia occurred within 48 h for reasons unrelated to the surgical site. Chemotherapy or radiotherapy initiated within 14 postoperative days was not an exclusion criterion and was examined separately.

### 2.5 Data Collection

Data were extracted from surgical, anaesthesia, pathology, laboratory, medication and follow‐up records using a standardised form. Variables included patient characteristics, body weight and condition score, reproductive and surgical history, ASA status, lesion characteristics, histopathology, procedure and wound class, operative and anaesthesia durations, temperature, antimicrobial exposure and documented rationale, closure practices, postoperative oncological treatment, follow‐up, SSI and microbiology. A random 20% sample was double‐checked against source records. Undocumented values were not imputed.

### 2.6 Follow‐Up

Outcome surveillance continued for at least 30 days. Primary follow‐up was classified as in‐person assessment, telephone follow‐up by clinic staff, mixed in‐person/telephone follow‐up, or subsequent medical‐record review when wound outcome was documented through later care. Reported wound abnormalities prompted review of subsequent available clinical documentation.

### 2.7 Surgical Site Infection Definition and Classification

The primary outcome was SSI identified during the prespecified 30‐day surveillance period. Each included record was screened for documented wound outcome and documented wound abnormalities prompted review of subsequent available clinical information. Because this was a retrospective study, SSI ascertainment relied on contemporaneous clinical and follow‐up documentation in the medical records. Records identified as SSI‐positive were independently re‐reviewed by two investigators using contemporary veterinary SSI terminology and classified as superficial incisional, deep incisional, or organ/space SSI according to the deepest documented tissue level involved (Verwilghen et al. 2026). Inter‐reviewer agreement for anatomical classification was assessed using Cohen's κ and disagreements were resolved by consensus. The contemporary veterinary consensus terminology was used to standardise retrospective classification and was not applied prospectively during clinical care. Evidence supporting SSI included compatible local inflammatory or wound findings, purulent or abnormal drainage, wound breakdown or dehiscence, abscess formation, compatible microbiology, or an attending veterinarian diagnosis supported by local findings; bacterial growth alone was insufficient. The 30‐day interval was used for outcome ascertainment and was not considered part of the biological definition of SSI. Definitions are summarised in Table 1 and Supplementary Table S2.

**TABLE 1:** Operational definitions for study outcomes and principal variables.

Variable	Operational definition
SSI	Infection following a surgical intervention involving superficial incisional, deep incisional and/or organ/space tissues opened or manipulated during that intervention; occurrence is independent of time.
30‐day surveillance period	Prespecified postoperative interval used to ascertain the primary outcome; not part of the biological SSI definition.
Superficial incisional SSI	SSI involving skin and/or subcutaneous tissue of the incision.
Deep incisional SSI	SSI involving fascia, muscle, or other deep incisional soft tissue.
Organ/space SSI	SSI involving an organ or anatomical space other than the incision that was opened or manipulated during surgery.
Deepest‐level rule	When more than one level was involved, classification used the deepest affected level.
MDR	Non‐susceptibility to at least one agent in three or more relevant antimicrobial categories.
Surgical duration	Time from incision to completion of closure.
Anaesthesia duration	Time from induction to extubation.
Intraoperative hypothermia	Any recorded intraoperative temperature < 36.5°C.
Complex mastectomy	Radical or bilateral mastectomy; simple and regional procedures were classified as standard.
Higher ASA status	ASA III–V versus ASA I–II.
Previous major surgery	Previous major abdominal, orthopaedic, hernia, tumour‐removal, or mammary procedure; OHE recorded separately.
Within‐species body weight	Body weight standardised separately within dogs and cats for the primary model.
Antimicrobial–isolate discordance	Subsequent isolate reported non‐susceptible to all relevant tested components of the recorded perioperative antimicrobial regimen, or outside the expected antibacterial spectrum of all agents in that regimen.

*Note*: SSI terminology and anatomical classification were standardised during retrospective re‐review using contemporary veterinary consensus terminology. The 30‐day interval was used for outcome ascertainment and was not considered part of the biological definition of SSI.

Abbreviations: ASA, American Society of Anesthesiologists; MDR, multidrug‐resistant; OHE, ovariohysterectomy; SSI, surgical site infection.

### 2.8 Histopathology and Indication for Non‐Malignant Mastectomy

Histopathology was classified as malignant, benign, or non‐neoplastic, with exact diagnoses retained for non‐malignant cases. The documented indication for surgery was extracted and malignant histopathology was evaluated as a potential SSI risk factor.

### 2.9 Reproductive and Previous Surgical History

Sex, reproductive status, previous and concurrent ovariohysterectomy and other previous major surgery were recorded separately. All 615 animals were female and were recorded as intact at the time of mastectomy; no previous or concurrent ovariohysterectomy was documented. Previous major surgery included major abdominal, orthopaedic, hernia, tumour‐removal, or mammary procedures. The documented rationale for not performing concurrent ovariohysterectomy was also extracted. Documentation that mastectomy was the sole planned procedure or that ovariohysterectomy was absent from the operative plan was distinguished from an explicitly documented clinical or owner‐related reason for not performing concurrent ovariohysterectomy.

### 2.10 Surgical and Perioperative Variables

Mastectomy was classified as simple, regional, radical, or bilateral; radical and bilateral procedures were categorised as complex in secondary analyses. Surgical duration was defined as the interval from incision to completion of closure and anaesthesia duration as the interval from induction to extubation. Intraoperative hypothermia was defined as any recorded intraoperative temperature < 36.5°C. Body condition score was recorded on a 9‐point scale. ASA status was grouped as I–II versus III–V. Glove and instrument changes before closure were recorded as yes, no, or undocumented.

### 2.11 Antimicrobial Exposure

Antimicrobial exposure was classified as preoperative prophylaxis, intraoperative administration/redosing, postoperative prophylaxis, or therapeutic treatment for suspected or confirmed SSI. Drugs were harmonised to generic active substances; preoperative timing and documented pre‐ and postoperative rationales were recorded. Because exposure was non‐randomised and likely influenced by perceived risk, associations with SSI were interpreted as observational rather than as estimates of prophylactic efficacy.

### 2.12 Microbiology and Antimicrobial Susceptibility

Culture reports were reviewed for specimen timing, aerobic and anaerobic culture, organism identification and susceptibility. Bacterial identification and susceptibility testing were performed by the diagnostic laboratories used by participating clinics. Where reports specified interpretive standards, Clinical and Laboratory Standards Institute veterinary guidance in use at the time was applied; otherwise, susceptibility categories were extracted as reported by the laboratory (Clinical and Laboratory Standards Institute 2024). Laboratory methods were not fully harmonised across clinics and years; therefore, microbiological results were analysed descriptively. Multidrug resistance (MDR) was assessed at the isolate level using the category‐based approach of Magiorakos et al. (2012). An isolate was classified as MDR when it was non‐susceptible to at least one tested agent in three or more relevant antimicrobial categories. Resistant and intermediate laboratory interpretations were treated as non‐susceptible. Intrinsic resistance was not counted as acquired resistance and untested agents or categories were not assumed to be either susceptible or non‐susceptible. For organism groups not explicitly covered by the original framework, the same ≥ 3‐category criterion was applied descriptively to the relevant antimicrobial categories represented in the available susceptibility report.

Where information permitted, the recorded perioperative antimicrobial regimen was compared with the subsequently identified organism and classified as susceptible/compatible, non‐susceptible, outside the expected antibacterial spectrum, or not assessable. When more than one perioperative antimicrobial had been administered, the complete recorded regimen was considered rather than selecting a single principal agent. An isolate was classified as susceptible/compatible when at least one component of the recorded regimen had documented susceptibility or expected antibacterial activity against the organism; as non‐susceptible when the organism was reported non‐susceptible to all relevant tested components; as outside the expected antibacterial spectrum when none of the recorded agents would ordinarily be expected to provide activity against the organism; and as not assessable when antimicrobial‐exposure or susceptibility information was insufficient for classification.

### 2.13 Clinic Infection‐Control Audit

The clinic‐level audit was a one‐time facility assessment. The audit was administered through an interview with the head veterinarian or clinic manager, followed by direct observation of surgical and waste‐handling areas. The same audit procedure was applied across all 12 participating clinics. A 10‐item descriptive audit was adapted from infection‐control domains within the World Health Organisation (WHO) Infection Prevention and Control Assessment Framework (World Health Organization 2018). Each affirmative item contributed one point, for a maximum score of 10.

Five additional variables—glove‐change protocol, instrument‐change protocol, aerobic‐culture availability, anaerobic‐culture availability and a written SAP protocol—were collected descriptively but were not scored. The adapted tool was not considered equivalent to or validated against the complete WHO framework. For descriptive presentation, audit scores were grouped as 0–3, 4–6, 7–8 and 9–10. These pragmatic author‐defined score bands were used only to summarise the adapted audit and should not be interpreted as validated WHO performance categories or as contemporaneous patient‐level exposures.

### 2.14 Statistical Analysis

Analyses were performed in *R* version 4.2. Continuous variables were summarised as mean ± standard deviation and/or median (IQR), as appropriate and categorical variables as counts and percentages. Distributions were assessed using histograms, quantile–quantile plots and Shapiro–Wilk tests; normality of predictors was not considered a prerequisite for logistic regression. SSI incidence was reported with a Wilson 95% confidence interval (CI). Univariable odds ratios (ORs) and multivariable adjusted odds ratios (aORs) were reported with 95% CIs, whereas principal exploratory sensitivity analyses were summarised using adjusted point estimates to assess consistency in direction and magnitude.

SSI associations were evaluated using clinic‐stratified conditional logistic regression, with clinic as the stratification factor; estimates therefore represent within‐clinic associations and strata without outcome variation do not contribute to the conditional likelihood. Continuous predictors were entered as linear terms on the log‐odds scale using the specified increments. The primary multivariable model was prespecified on clinical and literature grounds rather than univariable P‐value screening and included surgical duration per 30‐min increase, intraoperative hypothermia, body weight standardised within species, body condition score, ASA III–V status, malignant histopathology and previous major surgery.

Given 54 SSI events, model complexity was deliberately restricted. Largest tumour size and number of mammary masses were examined as secondary lesion‐burden variables and reported univariably but were not added post hoc to the primary model solely because of statistical significance. Anaesthesia duration and mastectomy complexity were evaluated in alternative models rather than simultaneously with surgical duration because they represent overlapping dimensions of operative burden; other recorded variables were examined in clinic‐stratified univariable or secondary analyses.

Sensitivity analyses were restricted to clean procedures, dogs, animals with at least one in‐person follow‐up and animals without chemotherapy/radiotherapy during the first 14 postoperative days. Preoperative, intraoperative and postoperative antimicrobial exposures were evaluated separately in clinic‐stratified univariable analyses. Closure practices were assessed in exploratory complete‐case clinic‐stratified models; glove‐ and instrument‐change status was undocumented in 52/615 procedures for each variable. Fisher's exact test was used for postoperative prophylaxis versus MDR among cultured SSI cases and for early postoperative chemotherapy/radiotherapy versus SSI. Tests were two‐sided with *P* < 0.05. Interpretation emphasised effect estimates and 95% CIs, while sensitivity analyses assessed consistency in direction and magnitude. All primary‐model variables were complete for all 615 procedures; no imputation was performed and missingness in exploratory closure‐practice variables is reported separately.

## 3 Results

### 3.1 Study Population and Follow‐up

During the study period, 748 potentially eligible mastectomy records were screened; 133 were excluded because of unavailable 30‐day outcome (*n* = 35), >20% missing key demographic, surgical and outcome variables (*n* = 32), concurrent major surgery (*n* = 28), preoperative chemotherapy/radiotherapy (*n* = 22), infection involving the surgical field (*n* = 11), or unrelated euthanasia within 48 h (*n* = 5). The final cohort comprised 615 procedures: 558 dogs (90.7%) and 57 cats (9.3%). Mean age was 10.2 ± 2.2 years; mean body weight was 24.2 ± 8.8 kg in dogs and 3.7 ± 1.1 kg in cats; and mean body condition score was 5.0 ± 1.1 on a 9‐point scale. Primary follow‐up was in person in 350 cases, by telephone in 140, mixed in‐person/telephone in 90 and by subsequent record review in 35. Overall, 440 animals had at least one in‐person assessment and telephone contact contributed to follow‐up in 230. Median follow‐up was 38 days (IQR, 33–42 days; range, 30–45 days). Cohort flow is shown in Figure 1.

**FIGURE 1:**
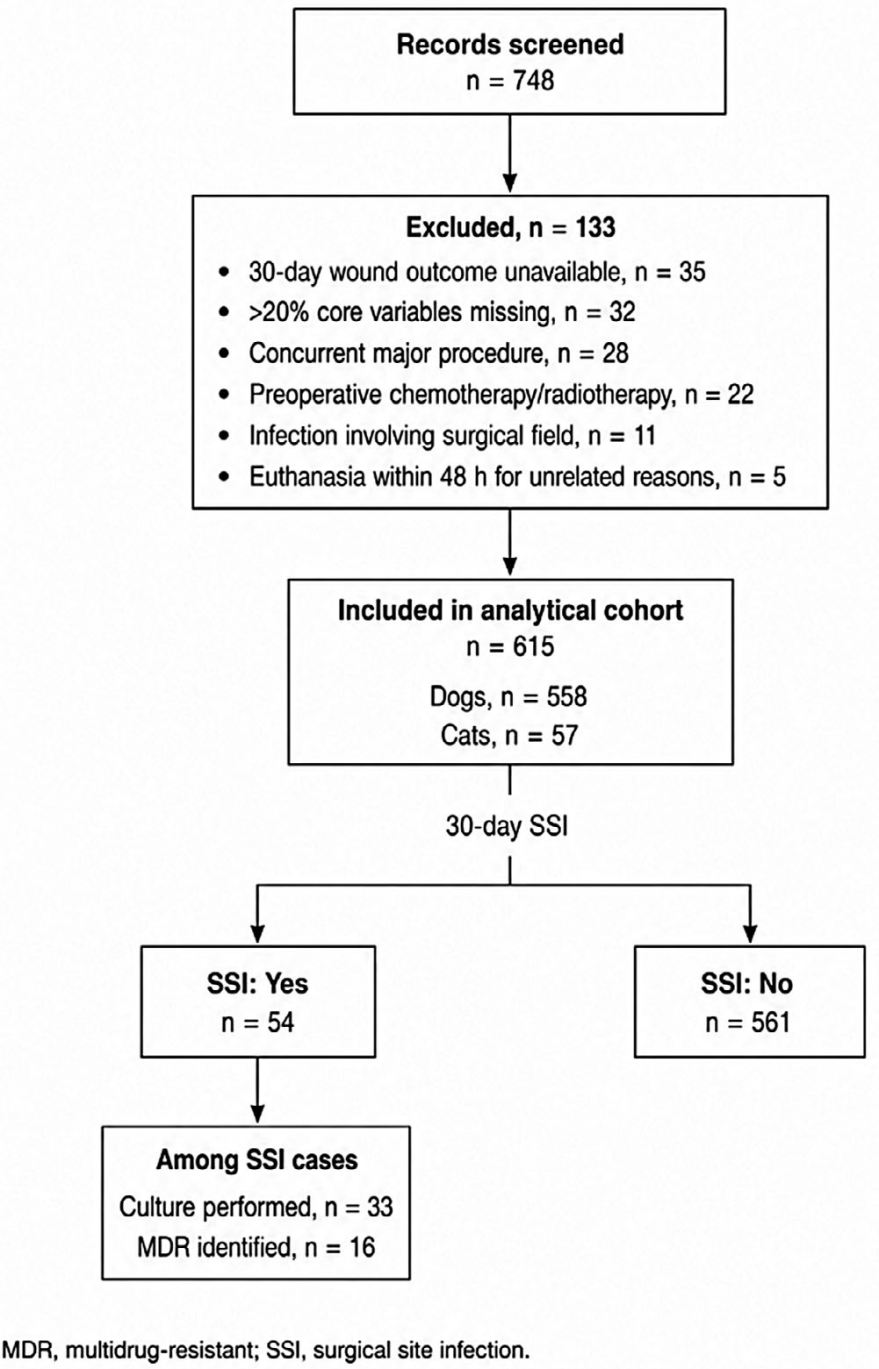
Study cohort selection and 30‐day surgical site infection outcomes. Of 748 potentially eligible records screened, 133 were excluded because the 30‐day wound outcome was unavailable (*n* = 35), more than 20% of key demographic, surgical and outcome variables were missing (*n* = 32), a concurrent major procedure was performed (*n* = 28), preoperative chemotherapy or radiotherapy had been administered (*n* = 22), infection involved the surgical field at the time of mastectomy (*n* = 11), or euthanasia occurred within 48 h for reasons unrelated to the surgical site (*n* = 5). The final cohort comprised 615 procedures (558 dogs and 57 cats); 54 developed SSI and 561 did not. Culture was performed in 33 SSI cases and an MDR isolate was identified in 16 of the 33 cultured SSI cases. Abbreviations: MDR, multidrug resistance; SSI, surgical site infection.

### 3.2 Surgical and Pathological Characteristics

Simple, regional, bilateral and radical mastectomy were performed in 234, 228, 111 and 42 cases, respectively; 153 procedures were classified as complex. Mean surgical duration was 94.3 ± 28.0 min (median, 89; IQR, 75–111) and mean anaesthesia duration was 147.0 ± 28.9 min (median, 143; IQR, 126–163.5). Intraoperative hypothermia occurred in 55 cases. Wound class was clean in 573, clean‐contaminated in 28 and contaminated in 14 procedures (Table 2). Histopathology was malignant in 411 cases, benign in 182 and non‐neoplastic in 22. The 204 non‐malignant diagnoses comprised benign mixed mammary tumour (*n* = 71), complex mammary adenoma (*n* = 37), simple mammary adenoma (*n* = 32), ductal adenoma (*n* = 19), fibroadenoma (*n* = 15), lobular mammary hyperplasia (*n* = 9), mammary adenoma (*n* = 8), chronic mastitis/inflammatory change (*n* = 5), duct ectasia (*n* = 4) and fibrocystic mammary change (*n* = 4). Among these cases, 86 underwent simple, 88 regional, 23 bilateral and seven radical mastectomy; indications are detailed in Supplementary Table S3.

**TABLE 2:** Baseline clinical, pathological and surgical characteristics.

Characteristic	Total, *N* = 615
Dogs	558 (90.7%)
Cats	57 (9.3%)
Female sex	615 (100.0%)
Intact reproductive status	615 (100.0%)
Age, years, mean ± SD	10.2 ± 2.2
Body weight, dogs, kg, mean ± SD	24.2 ± 8.8
Body weight, cats, kg, mean ± SD	3.7 ± 1.1
Body condition score, 9‐point scale, mean ± SD	5.0 ± 1.1
ASA I	111 (18.0%)
ASA II	319 (51.9%)
ASA III	148 (24.1%)
ASA IV	33 (5.4%)
ASA V	4 (0.7%)
Simple mastectomy	234 (38.0%)
Regional mastectomy	228 (37.1%)
Bilateral mastectomy	111 (18.0%)
Radical mastectomy	42 (6.8%)
Complex mastectomy	153 (24.9%)
Surgical duration, min, mean ± SD	94.3 ± 28.0
Surgical duration, median (IQR)	89 (75–111)
Anaesthesia duration, min, mean ± SD	147.0 ± 28.9
Anaesthesia duration, median (IQR)	143 (126–163.5)
Intraoperative hypothermia	55 (8.9%)
Clean procedure	573 (93.2%)
Clean‐contaminated	28 (4.6%)
Contaminated	14 (2.3%)
Malignant	411 (66.8%)
Benign	182 (29.6%)
Non‐neoplastic	22 (3.6%)
Previous major surgery	70 (11.4%)
Previous OHE	0 (0.0%)
Concurrent OHE	0 (0.0%)

*Note*: Data are *n* (%) unless otherwise specified. The 615 procedures represented 615 unique animals. Complex mastectomy comprised radical and bilateral procedures.

Abbreviations: ASA, American Society of Anesthesiologists; IQR, interquartile range; OHE, ovariohysterectomy; SD, standard deviation.

### 3.3 Reproductive and Previous Surgical History

All 615 animals were female and were recorded as intact at the time of mastectomy; no previous or concurrent ovariohysterectomy was documented. A specific reason for not performing concurrent ovariohysterectomy was recorded as owner decline after counselling in 126 cases and planned staged reproductive surgery in 102 cases. In the remaining 387 cases, records documented mastectomy as the sole planned procedure (*n* = 200), absence of ovariohysterectomy from the operative plan (*n* = 128), or no stated rationale (*n* = 59), without a distinct clinical or owner‐related reason. These findings describe historical documentation and should not be interpreted as evidence for or against concurrent ovariohysterectomy. Previous major surgery was recorded in 70 animals; SSI occurred in 11/70 animals with previous major surgery and 43/545 animals without previous major surgery.

### 3.4 Surgical Site Infection Incidence and Clinical Outcome

SSI was identified after 54/615 procedures (8.8%; Wilson 95% CI, 6.8%–11.3%): 50/558 dogs and 4/57 cats (Table 3). Fifty infections were classified as superficial incisional and four as deep incisional; no organ/space SSI was identified. Inter‐reviewer agreement for anatomical SSI classification was Cohen's κ = 0.83; disagreements were resolved by consensus. Median time to diagnosis was 7 days (IQR, 5–10). Five infected animals were readmitted, four underwent reoperation and two died within 30 days from causes documented as infection‐related.

**TABLE 3:** Thirty‐day surgical site infection outcomes.

Outcome	Result
SSI	54/615 (8.8%)
Wilson 95% CI	6.8%–11.3%
SSI in dogs	50/558 (9.0%)
SSI in cats	4/57 (7.0%)
Superficial incisional SSI	50/54 (92.6%)
Deep incisional SSI	4/54 (7.4%)
Organ/space SSI	0
Days to diagnosis, median (IQR)	7 (5–10)
Hospital readmission	5/54 (9.3%)
Reoperation	4/54 (7.4%)
Infection‐related 30‐day mortality	2/54 (3.7%)

*Note*: SSI was ascertained during the prespecified 30‐day postoperative surveillance period. Anatomical classification used the deepest documented tissue level involved.

Abbreviations: CI, confidence interval; IQR, interquartile range; SSI, surgical site infection.

### 3.5 Perioperative Antimicrobial Use

Preoperative prophylaxis was administered in 487/615 procedures; mean recorded timing was approximately 28 min before incision. Among recipients, a rationale was documented in 331 cases: anticipated extensive dissection (*n* = 149), clinic protocol (*n* = 104), or higher perceived patient/procedure risk (*n* = 78); no specific rationale was documented in 156 cases. Intraoperative administration/redosing occurred in 96/615 procedures. Postoperative prophylaxis was prescribed in 413/615 procedures, including 383/573 clean procedures, for a median of 5 days (IQR, 5–7). A rationale was documented in 213 cases: extensive dissection/dead‐space concern (*n* = 79), routine clinic practice (*n* = 65), prolonged procedure or higher ASA status (*n* = 42), large/ulcerated lesion (*n* = 14), or limited recheck access (*n* = 13); no specific rationale was documented in 200 cases (Table 4; Supplementary Table S4). Unadjusted SSI incidence was 8.2% versus 10.9% with versus without preoperative prophylaxis, 13.5% versus 7.9% with versus without intraoperative administration and 8.7% versus 8.9% with versus without postoperative prophylaxis. Clinic‐stratified univariable ORs were 0.59 (95% CI, 0.29–1.23; *P* = 0.160), 1.82 (95% CI, 0.85–3.89; *P* = 0.124) and 0.94 (95% CI, 0.49–1.81; *P* = 0.850), respectively (Supplementary Table S5). These observational estimates were not interpreted as measures of prophylactic efficacy.

**TABLE 4:** Perioperative antimicrobial exposure.

Characteristic	Result
Preoperative prophylaxis	487/615 (79.2%)
Cefazolin	186
Enrofloxacin	154
Amoxicillin–clavulanate	103
Gentamicin	24
Other/combination	20
Preoperative rationale documented	331/487 (68.0%)
Intraoperative administration/redosing	96/615 (15.6%)
Postoperative prophylaxis	413/615 (67.2%)
Postoperative duration, median (IQR)	5 (5–7) days
Postoperative rationale documented	213/413 (51.6%)

*Note*: Drug counts under preoperative prophylaxis refer to the recorded prophylactic regimen. Antimicrobial exposure was non‐randomised and likely influenced by clinicians’ perceptions of patient and procedural risk; these data should therefore not be interpreted as estimates of prophylactic efficacy. Detailed prescribing rationales are provided in Supplementary Table S4.

Abbreviation: IQR, interquartile range.

### 3.6 Microbiology and Antimicrobial Resistance

Culture was performed in 33/54 SSI cases; all 33 underwent aerobic culture and seven underwent additional anaerobic culture. Specimens were obtained before therapeutic antimicrobial administration in 19/54 SSI cases overall and in 19/33 cultured cases. A specific organism was recorded in 32/33 cultured cases; the remaining cultured record did not contain a specific organism identification. The recorded organisms were Escherichia coli (*n* = 12), Staphylococcus pseudintermedius (*n* = 10), Pseudomonas aeruginosa (*n* = 4), Klebsiella pneumoniae (*n* = 3), Enterococcus species (*n* = 2) and Proteus mirabilis (*n* = 1).

An MDR isolate was identified in 16/33 cultured SSI cases. MRSP was identified in 3/10 S. pseudintermedius isolates; 4/16 Enterobacterales were extended‐spectrum beta‐lactamase (ESBL)‐producing and 8/12 E. coli isolates were fluoroquinolone‐resistant. Antimicrobial–isolate comparison was assessable in 30/33 cultured SSI cases. Of the 30 assessable comparisons, 15/30 (50.0%) were susceptible/compatible with at least one component of the recorded perioperative antimicrobial regimen, 13/30 (43.3%) were non‐susceptible to all relevant tested components and 2/30 (6.7%) were outside the expected antibacterial spectrum of all agents in the regimen.

Thus, antimicrobial–isolate discordance was present in 15/30 (50.0%) assessable cases; 3/33 cultured cases were not assessable because antimicrobial‐exposure or susceptibility information was insufficient (Table 5; Supplementary Table S6). An MDR isolate was identified in 10/20 cultured SSI cases receiving postoperative prophylaxis and in 6/13 cultured SSI cases not receiving postoperative prophylaxis (Fisher's exact *P* = 1.000).

**TABLE 5:** Microbiology and antimicrobial resistance among cultured SSI cases.

Finding	Result
SSI cases cultured	33/54 (61.1%)
Aerobic culture performed	33/33 (100%)
Additional anaerobic culture performed	7/33 (21.2%)
Culture obtained before therapeutic antimicrobial administration	19/33 cultured SSI cases (57.6%); 19/54 SSI cases overall (35.2%)
Specific organism name available	32/33 (97.0%)
*Escherichia coli*	12
*Staphylococcus pseudintermedius*	10
*Pseudomonas aeruginosa*	4
*Klebsiella pneumoniae*	3
*Enterococcus* species	2
*Proteus mirabilis*	1
Cultured SSI cases with an MDR isolate	16/33 (48.5%)
MRSP	3/10 *S. pseudintermedius* isolates (30.0%)
ESBL‐producing Enterobacterales	4/16 (25.0%)
Fluoroquinolone‐resistant *E. coli*	8/12 (66.7%)
Assessable antimicrobial–isolate comparisons	30/33 (90.9%)
Susceptible/compatible with ≥1 component of perioperative regimen	15/30 (50.0%)
Non‐susceptible to all relevant tested components of perioperative regimen	13/30 (43.3%)
Outside expected antibacterial spectrum of all agents in perioperative regimen	2/30 (6.7%)
Overall antimicrobial–isolate discordance	15/30 (50.0%)
Antimicrobial–isolate comparison not assessable	3/33 (9.1%)
MDR among cultured SSI cases receiving postoperative prophylaxis	10/20 (50.0%)
MDR among cultured SSI cases not receiving postoperative prophylaxis	6/13 (46.2%)
Postoperative prophylaxis versus MDR	Fisher's exact *P* = 1.000

*Note*: Antimicrobial–isolate discordance was defined as an isolate being non‐susceptible to all relevant tested components of the recorded perioperative antimicrobial regimen or being outside the expected antibacterial spectrum of all agents in that regimen. When more than one perioperative antimicrobial had been administered, the complete recorded regimen was considered. Comparisons were classified as not assessable when antimicrobial‐exposure or susceptibility information was insufficient.

Abbreviations: ESBL, extended‐spectrum beta‐lactamase; MDR, multidrug‐resistant; MRSP, methicillin‐resistant Staphylococcus pseudintermedius; SSI, surgical site infection.

### 3.7 Early Postoperative Chemotherapy or Radiotherapy

Eighteen animals received chemotherapy or radiotherapy within 14 postoperative days. SSI occurred in 2/18 exposed and 52/597 unexposed animals (Fisher's exact *P* = 0.667). Excluding these animals did not materially change the principal estimates (Supplementary Table S7).

### 3.8 Glove and Instrument Change Before Closure

Gloves were documented as changed in 440 cases, not changed in 123 and undocumented in 52; corresponding instrument counts were 283, 280 and 52. Exploratory complete‐case clinic‐stratified model ORs were 0.77 (95% CI, 0.32–1.85; *P* = 0.566) for glove change and 0.87 (95% CI, 0.36–2.06; *P* = 0.743) for instrument change (Supplementary Table S8).

### 3.9 Infection‐Control Audit

All 12 clinics completed the audit. The mean adapted audit score was 4.6 ± 1.7/10; three clinics scored 0–3, seven scored 4–6, two scored 7–8 and none scored 9–10 (Table 6). Additional non‐scored practices are reported in Supplementary Table S9. Because the audit was a one‐time assessment, it was interpreted as descriptive context rather than as a causal exposure.

**TABLE 6:** Distribution of adapted infection‐control audit scores.

Score band	Clinics, *n* (%)
0–3	3 (25.0%)
4–6	7 (58.3%)
7–8	2 (16.7%)
9–10	0 (0.0%)
Audit score, mean ± SD	4.6 ± 1.7

*Note*: The score bands were pragmatic author‐defined groupings of an adapted 10‐item audit. Neither the adapted instrument nor the score bands were equivalent to or validated against the complete World Health Organisation Infection Prevention and Control Assessment Framework.

Abbreviation: SD, standard deviation.

### 3.10 Factors Associated With SSI

Clinic‐stratified univariable results are provided in Supplementary Table S5. Largest tumour size (OR, 1.39 per cm; 95% CI, 1.10–1.76; *P* = 0.006) and number of mammary masses (OR, 1.38 per additional mass; 95% CI, 1.14–1.66; *P* = 0.001) were associated with SSI in these secondary analyses. In the primary model, surgical duration per 30 min (aOR, 2.37; 95% CI, 1.61–3.49; *P* < 0.001), intraoperative hypothermia (aOR, 3.66; 95% CI, 1.39–9.62; *P* = 0.008), body condition score per point (aOR, 1.60; 95% CI, 1.05–2.42; *P* = 0.027) and previous major surgery (aOR, 3.57; 95% CI, 1.49–8.55; *P* = 0.004) were associated with SSI. Within‐species body weight (aOR, 0.73; 95% CI, 0.47–1.16; *P* = 0.183), ASA III–V status (aOR, 1.14; 95% CI, 0.55–2.36; *P* = 0.730) and malignant histopathology (aOR, 2.01; 95% CI, 0.91–4.44; *P* = 0.085) were not independently associated (Table 7; Figure 2). When surgical duration was replaced by mastectomy complexity, complex mastectomy had an aOR of 2.75 (95% CI, 1.31–5.80; *P* = 0.008). When surgical duration was replaced by anaesthesia duration, the aOR per 30‐min increase was 2.15 (95% CI, 1.48–3.13; *P* < 0.001). Associations for surgical duration, intraoperative hypothermia and previous major surgery were directionally consistent across the principal sensitivity analyses (Supplementary Table S7).

**TABLE 7:** Clinic‐stratified associations with surgical site infection.

Variable	Univariable OR (95% CI)	Adjusted OR (95% CI)	Adjusted P
Surgical duration, per 30 min	2.55 (1.81–3.60)	2.37 (1.61–3.49)	< 0.001
Intraoperative hypothermia	4.88 (2.14–11.12)	3.66 (1.39–9.62)	0.008
Weight, per within‐species SD	1.14 (0.84–1.56)	0.73 (0.47–1.16)	0.183
BCS, per point	1.35 (1.02–1.78)	1.60 (1.05–2.42)	0.027
ASA III–V	1.72 (0.91–3.25)	1.14 (0.55–2.36)	0.730
Malignant pathology	2.21 (1.07–4.58)	2.01 (0.91–4.44)	0.085
Previous major surgery	2.69 (1.17–6.19)	3.57 (1.49–8.55)	0.004

*Note*: Conditional logistic regression was stratified by clinic. Variables in the primary multivariable model were selected on clinical and literature grounds without univariable P‐value screening and all primary‐model variables were complete for the 615 procedures. Anaesthesia duration and mastectomy complexity were evaluated in separate alternative models (Supplementary Table S7) rather than entered simultaneously with surgical duration.

Abbreviations: ASA, American Society of Anesthesiologists; BCS, body condition score; CI, confidence interval; OR, odds ratio; SD, standard deviation; SSI, surgical site infection.

**FIGURE 2:**
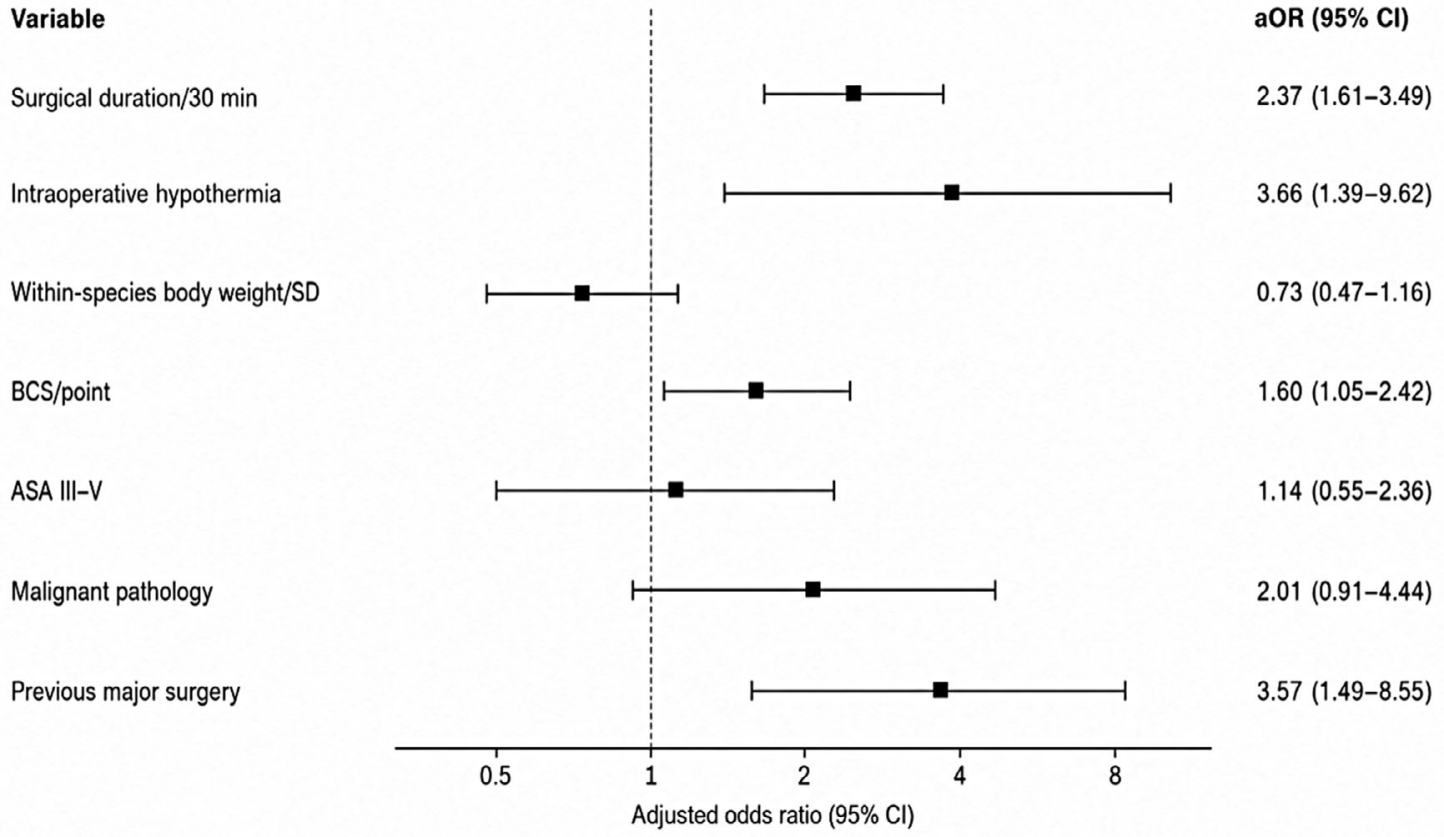
**Adjusted associations between clinical variables and surgical site infection**. Forest plot of adjusted odds ratios and 95% confidence intervals from the primary clinic‐stratified conditional logistic regression model. Surgical duration is expressed per 30‐min increase, body weight per within‐species standard deviation and body condition score per point; ASA III–V is compared with ASA I–II. Abbreviations: aOR, adjusted odds ratio; ASA, American Society of Anesthesiologists; BCS, body condition score; CI, confidence interval; SD, standard deviation; SSI, surgical site infection.

## 4 Discussion

SSI was identified after 8.8% of 615 mastectomy procedures; 50 infections were superficial incisional and four were deep incisional. In the primary clinic‐stratified model, longer surgical duration, intraoperative hypothermia, higher body condition score and previous major surgery were associated with SSI, whereas within‐species body weight, ASA status and malignant histopathology were not independently associated. Surgical duration, intraoperative hypothermia and previous major surgery remained directionally consistent in the principal sensitivity analyses.

The observed incidence was identical to the 8.8% reported after 376 canine mastectomies by Fuertes‐Recuero et al. (2025) and similar to the 8.9% reported by Spåre et al. (2021). Prolonged operative or anaesthetic duration has also been associated with SSI in other veterinary populations (Stetter et al. 2021; Fuertes‐Recuero et al. 2025). Alternative models using anaesthesia duration or mastectomy complexity likewise indicated that operative burden was associated with SSI. Body weight was not independently associated, despite its frequent identification as a risk factor after tibial plateau levelling osteotomy in a heterogeneous evidence base (Cavalli et al. 2026). ASA III–V status was also not independently associated, although an association has been reported in another small‐animal surgical population (Cavalli et al. 2025).

Largest tumour size and number of mammary masses were associated with SSI in secondary univariable analyses. Because these measures were not included in the clinically specified primary model, they should be interpreted as exploratory rather than as independent risk‐factor estimates. The 204 non‐malignant cases comprised 182 benign and 22 non‐neoplastic lesions treated for documented indications and malignant histopathology was not independently associated with SSI. Previous major surgery remained associated with SSI, but may represent cumulative healthcare exposure, underlying disease, altered anatomy, previous antimicrobial exposure, or greater clinical complexity rather than a direct causal effect. Current WSAVA guidelines recommend ovariohysterectomy at mammary tumour removal in intact bitches, except in cases of dedifferentiated tumours (Romagnoli et al. 2024). All animals in this cohort were intact and underwent mastectomy without concurrent ovariohysterectomy. The canine SSI incidence and associated risk‐factor findings therefore apply specifically to intact dogs undergoing mastectomy without concurrent ovariohysterectomy and may not generalise to dogs undergoing combined procedures. The reproductive‐history findings describe historical practice and do not support a recommendation for or against concurrent ovariohysterectomy.

Antimicrobial exposure was common: 79.2% of procedures received preoperative prophylaxis and 67.2% received postoperative prophylaxis, including 383/573 clean procedures. Contemporary ENOVAT guidance recommends against routine perioperative and postoperative SAP for clean soft‐tissue procedures other than neutering and its supporting meta‐analysis found generally trivial or small estimated benefits across the surgical categories evaluated (Allerton et al. 2026; Sørensen et al. 2026). Other studies likewise provide little support for routine prolonged postoperative prophylaxis in canine mastectomy or elective procedures (Spåre et al. 2021; Paeckel et al. 2024; Di Filippo et al. 2025). Because prescribing was non‐randomised and probably influenced by perceived risk, these exposure analyses do not estimate prophylactic efficacy.

The microbiological findings support culture‐guided treatment once SSI develops. An MDR isolate was identified in 16/33 cultured SSI cases and 15/30 assessable antimicrobial–isolate comparisons were discordant with the recorded perioperative regimen: 13 isolates were non‐susceptible to all relevant tested components and two were outside the expected antibacterial spectrum of all agents. These proportions apply only to the selected cultured subgroup. Anaerobic culture was performed in seven cultured cases and specimens were obtained before therapeutic antimicrobial administration in 19/33 cultured SSI cases (19/54 SSI cases overall); obligate anaerobes, polymicrobial infections and other organisms may therefore have been under‐detected (Williams et al. 2020).

Resistant organisms also have One Health relevance. Household sharing appears uncommon overall but is more frequent when an animal or owner has an active infection (Muturi et al. 2026) and closely related MRSP has been documented in an infected dog, household members and the attending surgeon (de Menezes et al. 2026). Owners, veterinary personnel and environmental sites were not sampled here and no molecular comparison was performed; transmission cannot be inferred.

The adapted clinic audit identified variation in formal infection‐control structures and practices. However, it was a one‐time assessment, was not temporally aligned with all procedures and used an author‐adapted, non‐validated score. The findings are therefore descriptive context rather than clinic‐level causal exposures or validated performance ratings.

This study has additional limitations. Its retrospective design made SSI ascertainment dependent on clinical and follow‐up documentation, so mild or incompletely documented infections may have been missed or misclassified. Exclusion of records with an unavailable 30‐day outcome or substantial missingness may have introduced selection bias if the probability of exclusion was related to postoperative infection; the direction and magnitude of this potential bias could not be determined. Follow‐up methods were heterogeneous, only 54 SSI events occurred, culture submission was selective, laboratory methods were incompletely harmonised and residual confounding is possible. The feline subgroup comprised only 57 cats with four SSI cases; consequently, the risk‐factor associations predominantly reflect the canine population and should not be interpreted as independently demonstrated in cats. Analyses of early postoperative oncological treatment and closure practices were limited by small exposed groups or incomplete documentation. The glove‐ and instrument‐change estimates should not be interpreted as evidence against established aseptic or oncological practice because these practices were neither randomised nor standardised. Continuous predictors were modelled as linear terms, so nonlinear relationships cannot be excluded. Results may be most applicable to comparable veterinary hospitals and clinics.

Prospective multicentre studies should use consensus SSI definitions, active standardised surveillance, harmonised microbiological methods and predefined perioperative protocols. Selected comparisons with previous veterinary SSI studies are summarised in Supplementary Table S10.

## 5 Conclusion

SSI was identified after 8.8% of canine and feline mastectomy procedures. Longer surgical duration, intraoperative hypothermia and previous major surgery showed the most consistent adjusted associations, while body condition score was associated in the primary model. These risk‐factor associations predominantly reflect the canine population and were not independently demonstrated in cats. The canine SSI incidence and associated risk‐factor findings apply specifically to intact dogs undergoing mastectomy without concurrent ovariohysterectomy and may not generalise to dogs undergoing combined procedures. Historical antimicrobial use was common; an MDR isolate was identified in 16/33 cultured SSI cases and antimicrobial–isolate discordance occurred in 15/30 assessable comparisons. These findings support standardised surveillance, perioperative risk reduction, culture‐guided treatment and contemporary antimicrobial stewardship.

## Author Contributions

**Mohammad Hedayatinia**: conceptualisation, methodology, investigation, data curation, project administration, resources, writing – original draft. **Arman Abdous**: conceptualisation, methodology, formal analysis, visualisation, validation, writing – original draft, writing – review and editing. **Ferdos Gorjian**: investigation, data curation, resources, validation, writing – review and editing. **Mobina Pato**: investigation, data curation, validation, resources, writing – review and editing. **Mohammad Ali Maadi**: methodology, validation, supervision, resources, writing – review and editing. **Reza Ahmadi**: investigation, validation, supervision, resources, writing – review and editing. All authors reviewed and approved the final revised manuscript and agreed to be accountable for all aspects of the work.

## Funding

The authors have nothing to report.

## Ethics Statement

The authors confirm that the ethical policies of *Veterinary Medicine and Science* have been adhered to. This study was approved by the Ethics Committee of Tehran University of Medical Sciences (IR.TUMS.SPH.REC.1400.396). The study involved retrospective review of de‐identified clinical records and a clinic‐level infection‐control audit. No experimental intervention was performed.

## Consent

The requirement for individual written owner consent for retrospective review of de‐identified clinical records was waived by the approving institution. When telephone follow‐up was undertaken, contact was conducted by clinic personnel using existing clinical records and owner‐identifying information was not transferred to the analytical dataset. Clinic managers provided permission for the infection‐control audit component.

## Conflicts of Interest

The authors declare no conflicts of interest.

## Supporting information




**Supporting File**: vms371322‐sup‐0001‐SuppMat.docx

## Data Availability

The de‐identified data supporting the findings are available from the corresponding author upon reasonable request, subject to institutional approval, participating‐clinic permission and applicable confidentiality restrictions.
