# Supplementary material for: Surgical Site Infection and Antimicrobial Resistance After Mastectomy in Dogs and Cats: A 615‐Case Multicentre Cohort and Infection‐Control Audit
Source: Vet Med Sci. 2026 Nov 3;12(6):e71322. doi: 10.1002/vms3.71322 (PMC13731168; doi:10.1002/vms3.71322)
Supplement: Supplementary file 1 [file VMS3-12-e71322-s001.docx]

# **SUPPORTING INFORMATION**

**Surgical Site Infection and Antimicrobial Resistance After Mastectomy in Dogs and Cats: A 615-Case Multicentre Cohort and Infection-Control Audit**

**Supplementary Table S1. STROBE-Vet checklist for the retrospective cohort component**

| **Item** | **No.** | **STROBE-Vet recommendation** | **Page/line reference** |
| --- | --- | --- | --- |
| **Title and Abstract** | **1(a)** | Indicate that the study was an observational study and, if applicable, use a common study design term | Title, lines 1–2; Abstract, Methods, lines 13–17; Section 2.2, lines 82–88. |
|  | **1(b)** | Indicate why the study was conducted, the design, the results, the limitations, and the relevance of the findings | Abstract, lines 3–31; limitations concerning reproductive status and feline inference are stated in the Abstract Conclusions, lines 26–31. |
| **Background / rationale** | **2** | Explain the scientific background and rationale for the investigation being reported | Introduction, lines 36–69. |
| **Objectives** | **3(a)** | State specific objectives, including any primary or secondary prespecified hypotheses or their absence | Introduction, objectives paragraph, lines 70–73; no formal prespecified hypotheses were stated. |
|  | **3(b)** | Ensure that the level of organizationᵃ is clear for each objective and hypothesis | Introduction, objectives paragraph, lines 70–73; Section 2.2, lines 82–88. |
| **Study design** | **4** | Present key elements of study design early in the paper | Abstract, Methods, lines 13–17; Sections 2.2–2.3, lines 82–93. |
| **Setting** | **5(a)** | Describe the setting, locations, and relevant dates, including periods of recruitment, exposure, follow-up, and data collection | Section 2.3, lines 90–93; Section 2.5, lines 105–111; Section 2.6, lines 113–117; Section 2.13, lines 193–206. |
|  | **5(b)** | If applicable, include information at each level of organization | Section 2.2, lines 82–88; Section 2.13, lines 193–206. |
| **Participantsᵇ** | **6(a)** | Describe the eligibility criteria for the owners/managers and for the animals, at each relevant level of organization | Animal eligibility: Section 2.4, lines 95–103. No owners/managers were enrolled as cohort participants; clinic-audit respondents are described in Section 2.13, lines 193–206. |
|  | **6(b)** | Describe the sources and methods of selection for the owners/managers and for the animals, at each relevant level of organization | Animal record source and selection: Sections 2.3–2.4, lines 90–103; Section 3.1, lines 246–257. Clinic-audit respondent and method: Section 2.13, lines 193–206; Section 3.9, lines 338–341. |
|  | **6(c)** | Describe the method of follow-up | Sections 2.6–2.7, lines 113–135. |
|  | **6(d)** | For matched studies, describe matching criteria and the number of matched individuals per subject (e.g., number of controls per case) | Not applicable; this was not a matched study. |
| **Variables** | **7(a)** | Clearly define all outcomes, exposures, predictors, potential confounders, and effect modifiers. If applicable, give diagnostic criteria | Section 2.5, lines 105–111; Sections 2.7–2.12, lines 119–191; Section 2.14, lines 216–230; Table 1; Supplementary Table S2. |
|  | **7(b)** | Describe the level of organization at which each variable was measured | Section 2.2, lines 82–88; Section 2.13, lines 193–206; Section 2.14, lines 216–223. |
|  | **7(c)** | For hypothesis-driven studies, the putative causal structure among variables should be described (a diagram is strongly encouraged) | Not described in the manuscript. |
| **Data sources / measurement** | **8*(a)** | For each variable of interest, give sources of data and details of methods of assessment (measurement). If applicable, describe comparability of assessment methods among groups and over time | Sections 2.5–2.13, lines 105–206; Table 1; Supplementary Table S2. |
|  | **8*(b)** | If a questionnaire was used to collect data, describe its development, validation, and administration | The patient-level component used retrospective records rather than a questionnaire (Section 2.5, lines 105–111). The clinic audit was administered by interview plus direct observation (Section 2.13, lines 193–206). |
|  | **8*(c)** | Describe whether or not individuals involved in data collection were blinded, when applicable | Not described in the manuscript. |
|  | **8*(d)** | Describe any efforts to assess the accuracy of the data (including methods used for “data cleaning” in primary research, or methods used for validating secondary data) | Section 2.5, lines 105–111; Section 2.7, lines 119–135; Section 2.14, lines 240–242; Section 3.4, lines 283–289. |
| **Bias** | **9** | Describe any efforts to address potential sources of bias due to confounding, selection, or information bias | Sections 2.4–2.5, lines 95–111; Section 2.11, lines 159–164; Section 2.14, lines 216–242; Discussion, lines 401–409 and 420–435. |
| **Study size** | **10(a)** | Describe how the study size was arrived at for each relevant level of organization | Sections 2.3–2.4, lines 90–103; Section 3.1, lines 246–250; Section 3.9, lines 338–341. All potentially eligible records during the study period were screened. |
|  | **10(b)** | Describe how non-independence of measurements was incorporated into sample-size considerations, if applicable | No formal sample-size calculation was performed. Each animal contributed one procedure (Section 2.2, lines 82–88), and clinic-level non-independence was addressed analytically using clinic-stratified regression (Section 2.14, lines 216–223). |
|  | **10(c)** | If a formal sample-size calculation was used, describe the parameters, assumptions, and methods that were used, including a justification for the effect size selected | Not applicable; no formal sample-size calculation was performed. Potentially eligible records from the study period were screened (Section 3.1, lines 246–250). |
| **Quantitative variables** | **11** | Explain how quantitative variables were handled in the analyses. If applicable, describe which groupings were chosen, and why | Section 2.10, lines 151–157; Section 2.13, lines 200–206; Section 2.14, lines 208–230. |
| **Statistical methods** | **12(a)** | Describe all statistical methods for each objective, at a level of detail sufficient for a knowledgeable reader to replicate the methods. Include a description of the approaches to variable selection, control of confounding, and methods used to control for non-independence of observations | Section 2.14, lines 208–242. |
|  | **12(b)** | Describe the rationale for examining subgroups and interactions and the methods used | Section 2.14, lines 224–242; no interaction analyses were reported. |
|  | **12(c)** | Explain how missing data were addressed | Sections 2.4–2.5, lines 95–111; Section 2.14, lines 231–242; Section 3.8, lines 333–336. |
|  | **12(d)** | If applicable, describe the analytical approach to loss to follow-up, matching, complex sampling, and multiplicity of analyses | Follow-up: Section 2.6, lines 113–117; clinic stratification: Section 2.14, lines 216–223; sensitivity and secondary analyses: Section 2.14, lines 231–242. No matching or complex sampling was used; no formal multiplicity adjustment was specified. |
|  | **12(e)** | Describe any methods used to assess the robustness of the analyses (e.g., sensitivity analyses or quantitative bias assessment) | Section 2.14, lines 231–242; Section 3.7, lines 329–331; Section 3.10, lines 353–357; Supplementary Table S7. |
| **Participants** | **13*(a)** | Report the numbers of owners/managers and animals at each stage of study and at each relevant level of organization – e.g., numbers eligible, included in the study, completing follow-up, and analysed | Section 3.1, lines 246–257; Section 3.9, lines 338–341; Figure 1 and its legend, lines 636–645. |
|  | **13*(b)** | Give reasons for non-participation at each stage and at each relevant level of organization | Section 3.1, lines 246–250; Figure 1 and its legend, lines 636–645. |
|  | **13*(c)** | Consider use of a flow diagram and/or a diagram of the organizational structure | Section 3.1, lines 256–257; Figure 1 and its legend, lines 636–645. |
| **Descriptive data on exposures and potential confounders** | **14*(a)** | Give characteristics of study participants (e.g., demographic, clinical, social) and information on exposures and potential confounders by group and level of organization, if applicable | Sections 3.1–3.3, lines 246–281; Table 2; Section 3.5, lines 291–300; Table 4. |
|  | **14*(b)** | Indicate number of participants with missing data for each variable of interest and at all relevant levels of organization | Section 2.5, lines 105–111; Section 2.14, lines 240–242; Section 3.8, lines 333–336. All primary-model variables were complete; glove- and instrument-change status was undocumented in 52/615 procedures for each variable; undocumented values were not imputed. |
|  | **14*(c)** | Summarize follow-up time (e.g., average and total amount), if appropriate to the study design | Section 3.1, lines 253–256. |
| **Outcome data** | **15*(a)** | Report outcomes as appropriate for the study design and summarize at all relevant levels of organization | Section 3.4, lines 283–289; Sections 3.6–3.9, lines 308–341; Tables 3, 5 and 6. |
|  | **15*(b)** | For proportions and rates, report the numerator and denominator | Sections 3.4–3.9, lines 283–341; Tables 3–6. |
|  | **15*(c)** | For continuous outcomes, report the number of observations and a measure of variability | The primary SSI outcome was binary. Time to diagnosis among SSI cases was summarised as median (IQR) in Section 3.4, lines 283–289, and Table 3. |
| **Main results** | **16(a)** | Give unadjusted estimates and, if applicable, adjusted estimates and their precision (e.g., 95% confidence interval). Make clear which confounders and interactions were adjusted. Report all relevant parameters that were part of the model | Section 2.14, lines 208–230; Section 3.10, lines 343–357; Table 7; Supplementary Table S5. |
|  | **16(b)** | Report category boundaries when continuous variables were categorized | Section 2.10, lines 151–157, for the intraoperative hypothermia threshold and ASA grouping; Section 2.13, lines 200–206, for audit score bands. |
|  | **16(c)** | If relevant, consider translating estimates of relative risk into absolute risk for a meaningful time period | Absolute 30-day SSI incidence is reported in Section 3.4, lines 283–289, and Table 3; absolute SSI proportions for antimicrobial-exposure comparisons are reported in Section 3.5, lines 301–303. |
| **Other analyses** | **17** | Report other analyses done, such as sensitivity/robustness analysis and analysis of subgroups | Section 2.14, lines 224–242; Section 3.5, lines 301–306; Sections 3.7–3.8, lines 329–336; Section 3.10, lines 353–357; Supplementary Tables S5, S7 and S8. |
| **Key results** | **18** | Summarize key results with reference to study objectives | Abstract, Results and Conclusions, lines 19–31; Discussion, opening paragraph, lines 359–365; Conclusion, lines 441–451. |
| **Strengths and Limitations** | **19** | Discuss strengths and limitations of the study, taking into account sources of potential bias or imprecision. Discuss both direction and magnitude of any potential bias | Discussion, lines 376–390, 401–409 and 416–435. |
| **Interpretation** | **20** | Give a cautious overall interpretation of results considering objectives, limitations, multiplicity of analyses, results from similar studies, and other relevant evidence | Discussion, lines 359–439; Conclusion, lines 441–451. |
| **Generalizability** | **21** | Discuss the generalizability (external validity) of the study results | Discussion, reproductive-history paragraph, lines 376–390; Discussion, limitations paragraph, lines 420–435; Conclusion, lines 441–451. |
| **Funding / Transparency** | **22(a)** | Funding – Give the source of funding and the role of the funders for the present study and, if applicable, for the original study on which the present article is based | Funding statement, line 460; separate Title Page, “Funding”. |
|  | **22(b)** | Conflicts of interest – Describe any conflicts of interest, or lack thereof, for each author | Conflict of Interest Statement, line 458; separate Title Page, “Conflict of Interest Statement”. |
|  | **22(c)** | Describe the authors’ roles – Provision of an authors’ declaration of transparency is recommended | Separate Title Page, pp. 2–3, “Author Contributions”. |
|  | **22(d)** | Ethical approval – Include information on ethical approval for use of animal and human subjects | Separate Title Page, “Ethics Approval Statement”; anonymised main manuscript, Section 2.1, lines 76–80. |
|  | **22(e)** | Quality standards – Describe any quality standards used in the conduct of the research | Section 2.2, lines 85–88; Section 2.5, lines 105–111; Section 2.7, lines 119–135; Section 2.12, lines 166–191; Section 2.13, lines 193–206; Section 2.14, lines 240–242. |

**Note**: The STROBE-Vet checklist was used for the retrospective cohort component. The clinic-level infection-control audit was a descriptive contextual component and was not treated as a patient-level causal exposure. **Abbreviation**: STROBE-Vet, Strengthening the Reporting of Observational Studies in Epidemiology—Veterinary extension.

**Supplementary Table S2. Operational definitions and coding**

| **Variable** | **Coding** |
| --- | --- |
| SSI | Infection following the surgical intervention involving superficial incisional, deep incisional, and/or organ/space tissues opened or manipulated during that intervention; occurrence is conceptually independent of time. |
| Surveillance period | 30 days after surgery. |
| Superficial incisional SSI | Involvement of skin and/or subcutaneous tissue. |
| Deep incisional SSI | Involvement of fascia, muscle, or deeper incisional soft tissue. |
| Organ/space SSI | Involvement of an organ or anatomical space opened or manipulated during surgery. |
| Deepest-level rule | Classified according to the deepest documented tissue level involved. |
| Culture-positive SSI | Clinically compatible SSI with bacterial growth; bacterial growth alone was insufficient. |
| MDR | Non-susceptibility to at least one agent in three or more relevant antimicrobial categories. |
| Surgical duration | Time from incision to completion of closure. |
| Anaesthesia duration | Time from induction to extubation. |
| Intraoperative hypothermia | Any recorded intraoperative temperature <36.5°C. |
| Standard mastectomy | Simple or regional mastectomy. |
| Complex mastectomy | Radical or bilateral mastectomy. |
| Higher ASA status | ASA III–V. |
| Malignant pathology | Malignant versus benign/non-neoplastic histopathology. |
| Body weight | Standardised within species in the primary model. |
| Previous major surgery | Previous major abdominal, orthopaedic, hernia, tumour-removal, or mammary procedure; OHE was recorded separately. |
| Early chemotherapy/RT | Chemotherapy or radiotherapy initiated within 14 postoperative days. |
| Culture before treatment | Specimen collected before administration of therapeutic antimicrobials. |
| Antimicrobial–isolate discordance | Subsequent isolate reported as non-susceptible to all relevant tested components of the recorded perioperative antimicrobial regimen, or outside the expected antibacterial spectrum of all agents in that regimen. |

**Note:** These operational definitions reflect the coding used in the analytical dataset. The 30-day surveillance interval was used for outcome ascertainment and was distinct from the biological definition of SSI.**Abbreviations:** ASA, American Society of Anesthesiologists; MDR, multidrug-resistant; OHE, ovariohysterectomy; RT, radiotherapy; SSI, surgical site infection.

## **Supplementary Table S3. Non-malignant pathology, procedure, and indication**

### **A. Exact non-malignant diagnoses**

| **Diagnosis** | **n** |
| --- | --- |
| Benign mixed mammary tumour | 71 |
| Complex mammary adenoma | 37 |
| Simple mammary adenoma | 32 |
| Ductal adenoma | 19 |
| Fibroadenoma | 15 |
| Lobular mammary hyperplasia | 9 |
| Mammary adenoma | 8 |
| Chronic mastitis/inflammatory change | 5 |
| Duct ectasia | 4 |
| Fibrocystic mammary change | 4 |
| **Total** | **204** |

### **B. Mastectomy type among non-malignant cases**

| **Type** | **Benign** | **Non-neoplastic** | **Total** |
| --- | --- | --- | --- |
| Simple | 76 | 10 | 86 |
| Regional | 80 | 8 | 88 |
| Bilateral | 19 | 4 | 23 |
| Radical | 7 | 0 | 7 |
| **Total** | **182** | **22** | **204** |

### **C. Documented indication for surgery**

| **Indication** | **n** |
| --- | --- |
| Progressive enlargement despite benign/indeterminate assessment | 70 |
| Multiple symptomatic mammary nodules | 38 |
| Ulceration or recurrent local irritation | 36 |
| Cytology/imaging unable to exclude malignancy | 28 |
| Persistent enlarging lesion with clinical concern | 11 |
| Owner elected definitive excision after counselling | 10 |
| Pain or recurrent inflammation | 6 |
| Indeterminate diagnosis requiring definitive excision | 3 |
| Recurrent enlargement affecting quality of life | 2 |
| **Total** | **204** |

**Note:** The non-malignant subgroup comprised 182 benign and 22 non-neoplastic cases. Sections A–C describe the exact histopathological diagnosis, mastectomy type, and documented clinical indication for the same 204 cases.**Abbreviations:** n, number of cases.

## **Supplementary Table S4. Antimicrobial use and documented rationale**

### **A. Preoperative antimicrobial**

| **Preoperative drug** | **n** |
| --- | --- |
| Cefazolin | 186 |
| Enrofloxacin | 154 |
| Amoxicillin–clavulanate | 103 |
| Gentamicin | 24 |
| Other/combination | 20 |
| No preoperative prophylaxis | 128 |

### **B. Preoperative rationale**

| **Documented preoperative rationale** | **n** |
| --- | --- |
| Anticipated extensive dissection | 149 |
| Clinic prophylaxis protocol | 104 |
| Higher perceived patient/procedure risk | 78 |
| No specific rationale documented | 156 |

### **C. Postoperative antimicrobial**

| **Postoperative drug** | **n** |
| --- | --- |
| Amoxicillin–clavulanate | 199 |
| Cephalexin | 109 |
| Enrofloxacin | 64 |
| Cefazolin | 20 |
| Other | 21 |
| No postoperative prophylaxis | 202 |

### **D. Postoperative rationale**

| **Documented postoperative rationale** | **n** |
| --- | --- |
| Extensive dissection/dead-space concern | 79 |
| Routine clinic practice | 65 |
| Prolonged procedure or higher ASA status | 42 |
| Large/ulcerated lesion | 14 |
| Limited access to recheck | 13 |
| No specific rationale documented | 200 |

**Note:** Preoperative prophylaxis was administered in 487 procedures and postoperative prophylaxis in 413 procedures. Sections B and D describe the rationale documented in the clinical record for these exposures. These historical prescribing rationales should not be interpreted as evidence-based indications or as estimates of prophylactic efficacy.**Abbreviations:** ASA, American Society of Anesthesiologists; n, number of procedures.

## **Supplementary Table S5. Clinic-stratified univariable associations with surgical site infection**

| **Variable** | **OR** | **95% CI** | **P** |
| --- | --- | --- | --- |
| Cat versus dog | 0.74 | 0.24–2.34 | 0.613 |
| Age, per 2 years | 1.04 | 0.78–1.37 | 0.804 |
| Weight, per within-species SD | 1.14 | 0.84–1.56 | 0.395 |
| Raw weight, per 5 kg | 1.07 | 0.92–1.25 | 0.365 |
| BCS, per point | 1.35 | 1.02–1.78 | 0.034 |
| ASA III–V | 1.72 | 0.91–3.25 | 0.095 |
| Malignant pathology | 2.21 | 1.07–4.58 | 0.033 |
| Largest tumour, per cm | 1.39 | 1.10–1.76 | 0.006 |
| Number of mammary masses, per additional mass | 1.38 | 1.14–1.66 | 0.001 |
| Previous major surgery | 2.69 | 1.17–6.19 | 0.020 |
| Surgical duration, per 30 min | 2.55 | 1.81–3.60 | <0.001 |
| Anaesthesia duration, per 30 min | 2.36 | 1.69–3.30 | <0.001 |
| Complex mastectomy | 3.62 | 1.87–7.03 | <0.001 |
| Intraoperative hypothermia | 4.88 | 2.14–11.12 | <0.001 |
| Non-clean wound | 1.42 | 0.43–4.65 | 0.563 |
| Preoperative prophylaxis | 0.59 | 0.29–1.23 | 0.160 |
| Intraoperative antimicrobial administration | 1.82 | 0.85–3.89 | 0.124 |
| Postoperative prophylaxis | 0.94 | 0.49–1.81 | 0.850 |
| Chemotherapy/radiotherapy within 14 postoperative days | 0.91 | 0.17–4.78 | 0.907 |

**Note:** ORs were estimated using clinic-stratified conditional logistic regression. Reference categories for binary variables were dog, ASA I–II, benign/non-neoplastic histopathology, no previous major surgery, standard mastectomy, no intraoperative hypothermia, clean wound classification, and absence of the specified antimicrobial or early oncological exposure, as applicable. Continuous-variable ORs are reported per increment shown.**Abbreviations:** ASA, American Society of Anesthesiologists; BCS, body condition score; CI, confidence interval; OR, odds ratio; RT, radiotherapy; SD, standard deviation; SSI, surgical site infection.

## **Supplementary Table S6. Microbiology and antimicrobial resistance**

| **Isolate** | **n** | **MDR, n** | **Fluoroquinolone resistant, n** |
| --- | --- | --- | --- |
| Escherichia coli | 12 | 6 | 8 |
| Staphylococcus pseudintermedius | 10 | 5 | 3 |
| Pseudomonas aeruginosa | 4 | 2 | 2 |
| Klebsiella pneumoniae | 3 | 2 | 2 |
| Enterococcus species | 2 | 1 | 1 |
| Proteus mirabilis | 1 | 0 | 0 |
| **Total named isolates** | **32** | **16** | **16** |

**Note:** A specific organism was identified in 32/33 cultured SSI cases. MRSP was identified in 3/10 S. pseudintermedius isolates, and 4/16 Enterobacterales were ESBL-producing. Aerobic culture was performed in 33/33 cultured SSI cases, and additional anaerobic culture was performed in 7/33. Cultures were obtained before therapeutic antimicrobial administration in 19/33 cultured SSI cases. Antimicrobial–isolate comparison was assessable in 30/33 cases: 15/30 (50.0%) were susceptible/compatible with at least one component of the recorded perioperative antimicrobial regimen, 13/30 (43.3%) were non-susceptible to all relevant tested components, and 2/30 (6.7%) were outside the expected antibacterial spectrum of all agents in the regimen. Overall antimicrobial–isolate discordance was therefore present in 15/30 (50.0%) assessable comparisons; 3/33 cases were not assessable because antimicrobial-exposure or susceptibility information was insufficient.**Abbreviations:** ESBL, extended-spectrum beta-lactamase; MDR, multidrug-resistant; MRSP, methicillin-resistant Staphylococcus pseudintermedius; n, number of isolates; SSI, surgical site infection.

## **Supplementary Table S7. Alternative and sensitivity models**

### **A. Alternative model replacing surgical duration with mastectomy complexity**

| **Variable** | **aOR** | **95% CI** | **P** |
| --- | --- | --- | --- |
| Complex mastectomy | 2.75 | 1.31–5.80 | 0.008 |
| Intraoperative hypothermia | 4.48 | 1.76–11.37 | 0.002 |
| Weight, per within-species SD | 0.77 | 0.49–1.20 | 0.244 |
| BCS, per point | 1.62 | 1.08–2.43 | 0.019 |
| ASA III–V | 1.47 | 0.74–2.94 | 0.272 |
| Malignant pathology | 1.79 | 0.82–3.89 | 0.143 |
| Previous major surgery | 3.26 | 1.38–7.69 | 0.007 |

### **B. Alternative model replacing surgical duration with anaesthesia duration**

| **Variable** | **aOR** | **95% CI** | **P** |
| --- | --- | --- | --- |
| Anaesthesia duration, per 30 min | 2.15 | 1.48–3.13 | <0.001 |
| Intraoperative hypothermia | 3.74 | 1.44–9.72 | 0.007 |
| Weight, per within-species SD | 0.74 | 0.47–1.16 | 0.187 |
| BCS, per point | 1.61 | 1.07–2.42 | 0.022 |
| ASA III–V | 1.13 | 0.54–2.35 | 0.747 |
| Malignant pathology | 2.03 | 0.92–4.49 | 0.079 |
| Previous major surgery | 3.34 | 1.40–7.98 | 0.007 |

### **C. Sensitivity analyses for the principal adjusted associations**

| **Analysis** | **Surgical duration, per 30 min, aOR** | **Intraoperative hypothermia, aOR** | **Previous major surgery, aOR** |
| --- | --- | --- | --- |
| Primary, N=615 | 2.37 | 3.66 | 3.57 |
| Clean procedures only, N=573 | 2.65 | 3.65 | 4.07 |
| Dogs only, N=558 | 2.26 | 4.14 | 3.97 |
| Excluding early chemotherapy/radiotherapy, N=597 | 2.39 | 3.77 | 3.87 |
| At least one in-person follow-up, N=440 | 2.63 | 3.47 | 3.18 |

**Note:** Sections A and B present alternative clinic-stratified multivariable models in which surgical duration was replaced by mastectomy complexity or anaesthesia duration, respectively. Section C presents adjusted point estimates for surgical duration, intraoperative hypothermia, and previous major surgery across the principal exploratory sensitivity analyses to summarise consistency in direction and magnitude. These sensitivity analyses were interpreted for robustness rather than statistical significance.**Abbreviations:** aOR, adjusted odds ratio; ASA, American Society of Anesthesiologists; BCS, body condition score; CI, confidence interval; N, number of procedures; RT, radiotherapy; SD, standard deviation.

## **Supplementary Table S8. Closure practices**

### **A. Documentation**

| **Variable** | **Yes** | **No** | **Undocumented** |
| --- | --- | --- | --- |
| Gloves changed before closure | 440 | 123 | 52 |
| Instruments changed before closure | 283 | 280 | 52 |

### **B. Exploratory complete-case clinic-stratified associations**

| **Variable** | **OR** | **95% CI** | **P** |
| --- | --- | --- | --- |
| Glove change | 0.77 | 0.32–1.85 | 0.566 |
| Instrument change | 0.87 | 0.36–2.06 | 0.743 |

**Note:** Section B presents exploratory complete-case clinic-stratified associations. These estimates should not be interpreted as evidence against established aseptic or oncological closure practices because documentation was incomplete and the practices were neither randomised nor standardised.**Abbreviations:** CI, confidence interval; OR, odds ratio.

## **Supplementary Table S9. Infection-control audit and additional contextual clinic-practice items**

### **A. Scored 10-item infection-control audit**

| **Audit item** | **Clinics meeting item, n/12** |
| --- | --- |
| IPC programme or responsible person | 5 |
| Written hand-hygiene protocol | 4 |
| Hand-hygiene compliance monitoring | 0 |
| Point-of-care alcohol-based hand rub | 9 |
| Infectious-waste segregation | 10 |
| Sharps safety containers | 12 |
| Surgical personal protective equipment | 12 |
| Routine culture before therapeutic antimicrobials | 3 |
| Complete written antimicrobial-prescribing protocol | 0 |
| AMR/IPC training in the preceding 12 months | 0 |

### **B. Additional contextual clinic-practice items not included in the audit score**

| **Additional item** | **Clinics meeting item, n/12** |
| --- | --- |
| Glove-change protocol before closure | 9 |
| Instrument-change protocol before closure | 5 |
| Routine aerobic culture available | 11 |
| Routine anaerobic culture available | 4 |
| Written surgical antimicrobial-prophylaxis protocol | 5 |

**Note:** Only the 10 items listed in section A contributed to the 10-point infection-control audit score. The five items listed in section B were collected for descriptive contextual purposes only and were not included in the audit score. The audit was a one-time facility assessment and was interpreted descriptively rather than as a contemporaneous causal exposure.**Abbreviations:** AMR, antimicrobial resistance; IPC, infection prevention and control; n, number of clinics.

## **Supplementary Table S10. Comparison with selected veterinary surgical site infection studies**

| **Study** | **Population** | **Main observation** | **Relevance** |
| --- | --- | --- | --- |
| Present study | 615 canine/feline mastectomies | SSI 8.8%; 92.6% superficial | Present cohort |
| Fuertes-Recuero et al., 2025 | 376 canine mastectomies | SSI 8.8%; 93.9% superficial | Closest procedure-specific comparator |
| Espinel-Rupérez et al., 2019 | Canine soft-tissue surgery | Prolonged surgery associated with SSI | Supports discussion of surgical duration |
| Husi et al., 2023 | 769 TPLO procedures | SSI 8.5%; resistant organisms identified among SSI cases | Orthopaedic comparison |
| Cavalli et al., 2026 | TPLO systematic review and meta-analysis | Increased body weight was the most frequently reported risk factor | Context for body-weight analysis |
| Di Filippo et al., 2025 | TPLO | Extended postoperative prophylaxis did not improve SSI outcome | Antimicrobial-stewardship context |
| Stutz et al., 2025 | Canine skin-tumour resections | Prospective comparison of prophylactic antimicrobial use | Clean soft-tissue comparator |
| Williams et al., 2020 | Gastrointestinal surgery | Postoperative wound organisms included E. coli | Microbiological comparison |
| Danieli et al., 2026 | Surgical and bite wounds | Resistant Gram-positive and Gram-negative organisms identified | AMR comparison |

**Note:** Studies were selected as clinically relevant comparators for SSI incidence, operative-duration risk, body-weight evidence, antimicrobial prophylaxis, and microbiology. Direct comparisons should be interpreted cautiously because the studies differed in surgical procedure, population, design, outcome ascertainment, and clinical setting.**Abbreviations:** AMR, antimicrobial resistance; GI, gastrointestinal; SSI, surgical site infection; TPLO, tibial plateau levelling osteotomy.

### **References for Supplementary Table S10**

Cavalli, S., Aragosa, F., Montano, C., Caterino, C., Della Valle, G., & Fatone, G. (2026). Assessment of risk factors for surgical site infection following tibial plateau levelling osteotomy: A systematic review and meta-analysis. Journal of Small Animal Practice. Advance online publication. doi:10.1111/jsap.70133.

Danieli, D., Amadori, M., Crimi, S., Pregnolato, F., Caruso, C., Gambino, G., et al. (2026). Antimicrobial resistant isolates in surgical and bite wounds in dogs and cats: A 12-year retrospective analysis. Animals, 16(3), 501. doi:10.3390/ani16030501.

Di Filippo, L., Bianchi, A., Paolini, A., Maggiolini, U., Niebauer, G. W., Collivignarelli, F., et al. (2025). Comparison of surgical site infection rates in dogs undergoing tibial plateau leveling osteotomy using perioperative versus peri- and postoperative antimicrobial prophylaxis. Veterinary Sciences, 12(3), 258. doi:10.3390/vetsci12030258.

Espinel-Rupérez, J., Martín-Ríos, M. D., Salazar, V., Baquero-Artigao, M. R., & Ortiz-Díez, G. (2019). Incidence of surgical site infection in dogs undergoing soft tissue surgery: Risk factors and economic impact. Veterinary Record Open, 6(1), e000233. doi:10.1136/vetreco-2017-000233.

Fuertes-Recuero, M., Penelo, S., Suarez-Redondo, M., Eceiza-Zubicaray, A., Arenillas, M., Valdivia, G., et al. (2025). Incidence and risk factors of surgical site infection in 376 mastectomy procedures in female dogs: A retrospective cohort study. Veterinary Sciences, 12(6), 553. doi:10.3390/vetsci12060553.

Husi, B., Overesch, G., Forterre, F., & Rytz, U. (2023). Surgical site infection after 769 tibial plateau leveling osteotomies. Frontiers in Veterinary Science, 10, 1133813. doi:10.3389/fvets.2023.1133813.

Stutz, Z. J., Kowalska, M. E., Degen, M., & Nolff, M. C. (2025). Impact of perioperative prophylactic antibiotics on surgical site infection rate after skin tumor removals in dogs: A prospective observational study. Tierärztliche Praxis Ausgabe K: Kleintiere/Heimtiere, 53(5), 274–279. doi:10.1055/a-2685-0759.

Williams, R. W., Cole, S., & Holt, D. E. (2020). Microorganisms associated with incisional infections after gastrointestinal surgery in dogs and cats. Veterinary Surgery, 49(7), 1301–1306. doi:10.1111/vsu.13495.
